# Comparison of the effects of vitamin D products in a psoriasis plaque test and a murine psoriasis xenograft model

**DOI:** 10.1186/1479-5876-7-107

**Published:** 2009-12-17

**Authors:** Peter H Kvist, Lars Svensson, Oskar Hagberg, Vibeke Hoffmann, Kaare Kemp, Mads A Røpke

**Affiliations:** 1 Department of Disease Pharmacology, LEO Pharma A/S, Industriparken 55, DK-2750 Ballerup, Denmark; 2 Department of Biostatistics, LEO Pharma A/S, Industriparken 55, DK-2750 Ballerup, Denmark; 3 Department of Clinical Operations, LEO Pharma A/S, Industriparken 55, DK-2750 Ballerup, Denmark; 4 Translational Research, LEO Pharma A/S, Industriparken 55, DK-2750 Ballerup, Denmark

## Abstract

The aim of the present study was to compare the effects of Daivobet^® ^and calcipotriol on clinical score and biomarker responses in a modified version of the Scholtz-Dumas psoriasis plaque assay. Furthermore, it was the aim to compare the effects of calcipotriol and betamethasone in the murine psoriasis xenograft model. Twenty four patients with psoriasis were treated topically once daily for three weeks, whereas the grafted mice were treated for four weeks. Clinical responses were scored twice weekly and biopsies were taken at the end of each study to analyse for skin biomarkers by histology and immunohistochemistry. The results clearly demonstrate effects on both clinical signs and biomarkers. In the patient study the total clinical score was reduced significantly with both Daivobet^® ^and calcipotriol. Both treatments reduced epidermal thickness, Ki-67 and cytokeratin 16 expression. T cell infiltration was significantly reduced by Daivobet^® ^but only marginally by calcipotriol. Both treatments showed strong effects on the epidermal psoriatic phenotype.

Results from the xenograft model essentially showed the same results. However differences were observed when investigating subtypes of T cells.

The study demonstrates the feasibility of obtaining robust biomarker data in the psoriasis plaque test that correlate well with those obtained in other clinical studies. Furthermore, the biomarker data from the plaque test correlate with biopsy data from the grafted mice.

## Background

Psoriasis is a common skin disease characterized by increased inflammation as well as increased proliferation and altered differentiation of keratinocytes, resulting in characteristic plaques on the skin [1]. The complexity of this disease and the fact that the structure of human skin is very different from most animals has made it very challenging to mimic human psoriasis in preclinical models. In the search for new effective topical treatments of psoriasis it is therefore important to be able to get early clinical "proof-of-concept" in psoriasis patients as well as an understanding of the mechanism of action as early as possible. This also enables early discontinuation of the development of non-effective compounds. One possibility is the use of experimental clinical models such as the psoriasis plaque test, originally developed by Scholtz and Dumas [2], which allows simultaneous topical treatment with several active compounds and controls in the same psoriasis patient. The psoriasis xenograft SCID mouse model is probably the most relevant animal model for efficacy testing of novel anti-psoriatic drugs [3]. In this model, keratome biopsies from psoriatic plaques are transferred to the back of SCID mice and the mice are subsequently treated with compounds either systemically or topically. The model has been used for several years and is recognized as predictive for the outcome in clinical trials. However, it is still debated which endpoints are relevant and to what extent the investigation of biomarkers in this model is meaningful.

In the present study we compare the effects of ointment vehicle, calcipotriol ointment and calcipotriol plus betamethasone dipropionate (BDP) ointment (Daivobet^®^) on the clinical score in a psoriasis plaque test as well as the effect on skin biomarkers, both in the clinical setting and the preclinical psoriasis model.

## Materials and methods

### Patients and design

Twenty-four patients with stable chronic plaque-type psoriasis were included in this study after the relevant Independent Ethic Committee gave its approval and the patients gave their signed informed consents. The clinical investigation was conducted according to Declaration of Helsinki principles and Good Clinical Practice. The study was a single centre, investigator blinded, within-subject randomised, active- and vehicle-controlled, repeated dose study, conducted at CPCAD, Nice, France. No topical treatment had been applied for four weeks prior to admission and none of the patients had received systemic treatment for their psoriasis within 12 weeks prior to the study.

The study was conducted as a modified version of the psoriasis plaque test derived from the method described by KJ Dumas and JR Scholtz [2]. For each subject, six test sites of 2-cm diameter were selected on predetermined lesions, and a circular adhesive device was placed on each site. The study medications were applied six times a week (once daily Monday to Saturday) for three weeks, using an Eppendorf^® ^combitip and they were rubbed into the lesions using a gloved finger. The test sites were then covered with an unocclusive gaze and the system was secured on the skin using a Tegaderm^® ^(3 M, Cergy-Pontoise Cedex, France) dressing with a hole at the centre. The test areas were randomised and treated with Daivobet^® ^ointment (calcipotriol 50 μg/g plus betamethasone 0.5 mg/g as diprosone), calcipotriol ointment (50 μg/g), three experimental formulations and ointment vehicle.

Clinical rating was performed twice a week during the treatment phase assessing the Total Clinical Score (TCS). The Total Clinical Score is defined as the sum of erythema (0-3), scaling (0-3) and thickness (0-3) scores. Total Clinical Scores therefore range from 0 (all symptoms absent) to 9 (all symptoms severe).

At the end of the treatment (on the day after the last treatment) all subjects had two 4 mm punch biopsies taken from two of the three sites treated with Daivobet^® ^ointment, the calcipotriol ointment and ointment vehicle. A biopsy randomisation was put in place to ensure that an equal amount of biopsies was taken from each of these three treatments. The biopsies were fixed in buffered formalin immediately after sampling and fixed for at least 24 hrs before being embedded in paraffin.

### Sampling of biopsies for the psoriasis xenograft SCID mouse model

Patients suffering from chronic plaque-psoriasis were used as donors for the psoriatic keratome biopsies. The removal of skin and subsequent experiments were approved by the local ethical committees and the patients gave their signed informed consent. Patients were locally anesthetized and psoriatic keratome biopsies (thickness 0.5 mm) were removed using a dermatome shaver. Three keratome biopsies (containing both dermis and epidermis) were obtained after informed consent from four psoriasis patients. Biopsies were taken from infiltrated red plaques located on the anterior or lateral aspect of the femoral region.

### Grafting of mice

The keratome biopsy from each patient was divided into pieces of 1.5 × 1.5 cm. As recipients, female CB.17 SCID mice (M&B Taconic, Denmark) aged 6 weeks were used. The mice were anesthetized using a mixture of Ketamin (Ketaminol Vet, Intervet, Denmark) and Xylazin (Rompun Vet, Bayer A/S, Denmark) and a fully excision biopsy was removed from the back of the animals. The split human keratome biopsies were then grafted onto the back of the animals. The grafts were protected by a bandage during the following two weeks. All the transplantation procedures were performed under semi-sterile conditions. The animals were stored in special-pathogen-free (SPF) environment during entire experiment. The experiments were carried out in accordance with the local ethics committee and with animal welfare guidelines provided by the Animal Experiments Inspectorate, Ministry of Justice, Denmark.

### Treatment of animals

After two weeks of rest, the animals were randomized into three groups. The groups were treated with the calcipotriol ointment (calcipotriol, 50 μg/g) (n = 7), betamethasone (0.5 mg/g (as diproprionate; BDP) in ointment (n = 4) or ointment vehicle (n = 5) twice daily.

After 4 weeks of treatment, the animals were bled and sacrificed, and a 4-mm punch biopsy was taken from each xenograft. Biopsies were fixed in 10% neutral buffered formalin for a maximum of 48 hours, were processed according to standard histological procedures and embedded in paraffin. Tissue sections were mounted on adhesive slides (Superfrost^® ^Plus, MENZEL-GLASER, Germany) and stained with hematoxylin and eosin (H&E, Merck, Darmstadt, Germany). Immunohistochemical stainings were performed as described under *Immunohistochemistry*.

### Immunohistochemistry

The following markers were investigated: (i) T cell infiltration: CD3, CD4, CD8, CD45RO; (ii) epidermal differentiation: fraction of cytokeratin 10 (CK10) and cytokeratin 16 (CK16) positive epidermis (iii); epidermal proliferation: Ki-67-positive keratinocytes.

The tissue blocks were sectioned into 3-4 μm sections, mounted on adhesive slides (Superfrost^® ^Plus, MENZEL-GLÄSER, Germany) and kept at 4°C until processed.

Prior to staining the tissue sections were deparaffinized and rehydrated and then incubated in hydrogen peroxide (3%) for 5 min to quench endogenous peroxidase (optimal immunohistochemical staining of CD4 required blocking of endogenous HRP after incubation of the primary antibody).

Sections were then submitted to heat induced epitope retrieval by incubation in boiling Tris-EGTA (T-EG) buffer (pH 9) in a microwave oven for 15 min. This was followed by incubation in the T-EG buffer for 15 min at room temperature (RT), and afterwards by 5 min incubation in TBS/Tween (LAB42006, Bie & Berntsen, Rødovre, Denmark). All subsequent incubations were performed on a DAKO Autostainer (Dako Autostainer Plus, DAKO, Glostrup, Denmark) at RT. The slides were mounted into the autostainer and washed in Wash Buffer (S3006), after which unspecific protein binding was blocked by incubation in 10% goat serum (X0907, DAKO, Glostrup, Denmark) for 10 min. All slides were incubated with primary antibody diluted in Antibody Diluent (S2022, DAKO, Glostrup, Denmark) at different concentrations for 1 h. The following primary antibodies were used: anti-CD3 (polyclonal; 2 mg/L), anti-CD8 (clone C8/144B, 2 mg/L), anti-CD45RO (clone UCHL1; 4,4 mg/L), anti-CK10 (clone DE-CK10, 1 mg/L), anti-Ki-67 (clone MIB-1, 0,5 mg/L), all obtained from DAKO, Glostrup, Denmark, anti-CK16 (clone LL025, 2,5 mg/L) obtained from AbD Serotec, Scandinavia and anti-CD4 (clone 1F6, 2 mg/L) obtained from NovoCastra, UK.

The detection systems EnVision+ for rabbit antibodies (K4003, DAKO, Glostrup, Denmark) and EnVision for mouse antibodies (K4001, DAKO, Glostrup, Denmark) were applied according to the manufacturers' instructions. Slides were stained with liquid diaminobenzidine tetrahydrochloride (DAB+), a high-sensitivity substrate-chromogen system (K3468, DAKO, Glostrup, Denmark). Counterstaining was performed with Meyer's haematoxylin. The sections were washed in tap and distilled water and mounted with Pertex. Control immunohistochemical stainings were run on parallel sections without the primary antibody and with a nonsense polyclonal or monoclonal (matching isotype) antibody at same concentration as the primary antibody.

### Immunohistochemical evaluation

The immunohistochemical stainings were assessed on an Olympus BX51 light microscope coupled to a computer equipped with Microimager software. All positive cells in one representative and blinded tissue section were counted.

In the evaluation of staining for CD3, CD4, CD8, CD45RO, only cells with staining restricted to the plasma membrane and a visible nucleus were counted as positive. Cells in the epidermis and the 200 μm of dermis below the basement membrane were counted with a 20× objective and the numbers were reported per mm^2^. In the evaluation of staining for Ki67, only epidermal cells with nuclear staining were counted as positive and reported as No/mm (surface length). The distribution of CK10 and CK16 in epidermis was measured by absolute numbers using the fraction of the positively stained epidermal area, i.e. the area of positive CK10 or CK16 in relation to the total epidermal area. These measurements were performed with the Visiopharm Integrator System software (VIS, Visiopharm, Hørsholm, Denmark) on serial sections.

### Histopathological evaluation

Haematoxylin and eosin (HE) stained sections from the plaque test study were evaluated by a pathologist in a blinded fashion. The following parameters were measured in absolute numbers or scored semi-quantitatively using a 0-3 scale: Epidermal thickness, thinning (absence) of stratum corneum, extent of stratum granulosum, extent of parakeratosis, extent of inflammatory cell infiltration and frequency of neutrophil microabscesses.

### Statistics

*Clinical data: *Since the effects of three treatments were analysed based on only two biopsies per patient the statistical analysis was based on data in an incomplete block structure. The study and statistical design was chosen so as to make the data as balanced as possible. P-values were calculated with standard assumptions about independence, equivariance and normality. Due to heteroscedacity, the logarithm of the following response variables was used: CD3, CD4, CD45, and Ki-67. *SCID mouse data: *Data from the SCID mice were analysed with a two-sample t-test with Welch-Satterthwaite approximation for the degrees of freedom. All P-values were computed using the R package (R Development Core Team, 2008).

## Results

### Patient study

**Figure 1:**
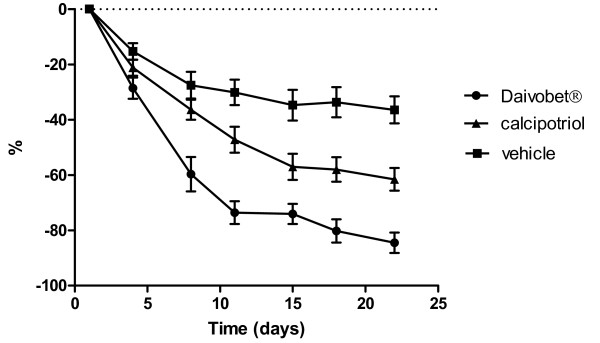
**Percent reduction (mean ± SEM, n = 12) in total clinical score (TCS) from day 1 to day 22 after treatment with Daivobet^® ^vehicle, calcipotriol and Daivobet^® ^once daily 6 times weekly (excl. Sunday) for three weeks**.

**Figure 2:**
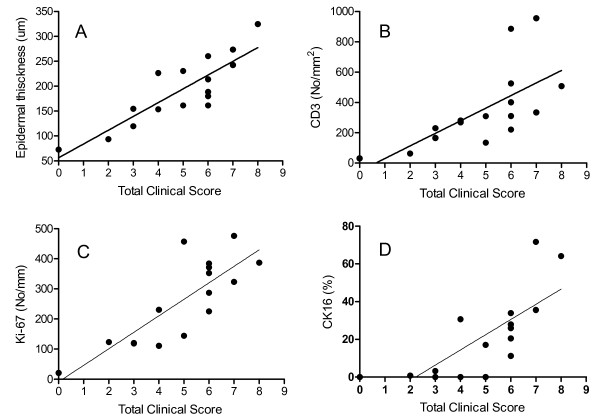
**Correlation between biomarker responses and total clinical score in skin samples from psoriasis patients treated for three weeks with ointment vehicle**. Correlation of clinical score with epidermal thickness (A; r = 0.733), CD3 positive T cells (B; r = 0.375), Ki-67 positive cells (C; r = 0.675) or cytokeratin 16 positive cells (D; r = 0.628).

The clinical and biomarker scores are shown in Figure 1 and Table 1, respectively, and the correlation between total clinical score (TCS) and biomarkers is shown in Figure 2. The mean reductions in TCS between day 0 and day 21 was 2.71 (44%), 4.48 (73%) and 6.19 (100%) for the ointment vehicle, calcipotriol ointment and Daivobet^® ^ointment groups, respectively. The reduction induced by calcipotriol was statistical significant (p < 0.001) compared to vehicle at day 21. Daivobet^® ^reduced TCS significantly compared to both vehicle (p < 0.001) and calcipotriol (p < 0.001). The reduction was statistical significant (p < 0.05) from day 4 for Daivobet^® ^and at day 11 for calcipotriol.

**Table 1:** Immunohistochemical and histopathological scores obtained from skin biopsies from the psoriasis plaque test with treatments of vehicle, calcipotriol and Daivobet^® ^ointment.

	Vehicle	calcipotriol		Daivobet^®^		
	n = 16	n = 16	p-value vs. vehicle	n = 16	p-value vs. vehicle	p-value vs. calcipotriol
CD3 (number/mm^2^)	351.7 ± 261.0	309.0 ± 199.0	NS	97.6 ± 72.5	<0.001	<0.001
CD4 (number/mm^2^)	347.9 ± 221.7	289.2 ± 128.9	NS	104.2 ± 69.1	0.001	0.002
CD8 (number/mm^2^)	156.0 ± 111.7	152.4 ± 113.3	NS	44.5 ± 30.2	<0.001	<0.001
CD45RO (number/mm^2^)	419.0 ± 284.4	329.9 ± 201.7	NS	96.1 ± 70.4	<0.001	<0.001
Ki-67 (number/mm)	258.5 ± 140.5	165.8 ± 107.5	NS	43.2 ± 25.7	<0.001	<0.001
CK10 (% area)	64.2 ± 9.3	65.5 ± 10.7	NS	68.8 ± 13.3	NS	NS
CK16 (% area)	21.4 ± 22.5	11.4 ± 15.7	NS	0.1 ± 0.4	<0.001	0.028
Epidermal thickness (μm)	191.0 ± 67.6	148.8 ± 63.4	0.01	66.5 ± 15.3	<0.001	<0.001
Strat corneum (extent)	2.1 ± 1.0	1.0 ± 0.8	0.002	0.2 ± 0.4	<0.001	0.005
Strat granulosum (extent)	1.4 ± 1.4	0.4 ± 0.9	0.007	0.0 ± 0.0	<0.001	NS
Parakeratosis (extent)	1.6 ± 1.3	0.5 ± 0.9	0.002	0.0 ± 0.0	<0.001	0.04
Infiltration (extent)	1.5 ± 0.6	1.2 ± 0.4	NS	0.9 ± 0.3	<0.001	NS

NS, not significant.

**Figure 3:**
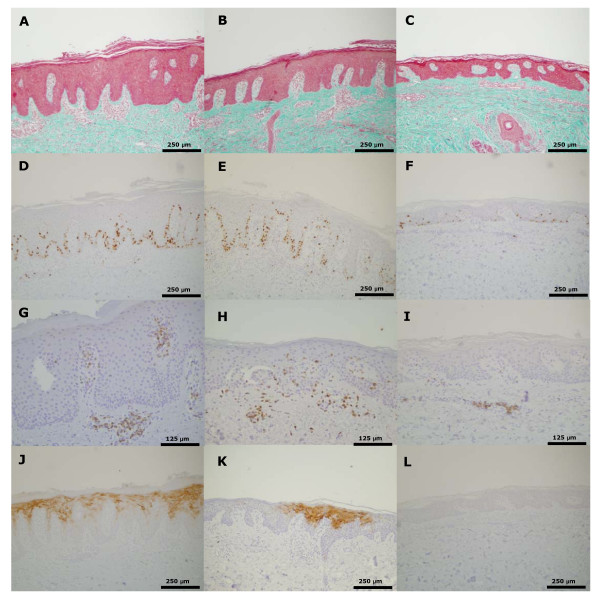
**Biomarker endpoints after treatment with vehicle (A, D, G and J), calcipotriol (B, E, H and K) and Daivobet^® ^(C, F, I and L) in the psoriasis plaque assay**. A-C: Masson-Trichrome (MT) staining showing epidermal hyperplasia. D-F: Proliferation of keratinocytes in stratum basale of epidermis (Ki-67). G-I: Immunohistochemical staining with a pan-T cell marker (CD3). J-L: Epidermal expression of keratin 16 show decrease after treatment with calcipotriol and is absent after treatment with Daivobet^®^.

Daivobet^® ^induced a highly significant reduction in epidermal thickness compared to vehicle (72%) and an almost complete normalisation of the epithelial morphology (Table 1 and Figure 3). Stratum granulosum was normalized and parakeratosis was absent in all samples treated with Daivobet^®^. This was supported by the lack of CK16 staining in these samples. Furthermore, the number of Ki-67 positive epidermal cells was reduced by 83% compared to vehicle treated skin. The correlation of epidermal thickness, proliferative activity (Ki-67 expression) and cytokeratin 16 expression with TCS is shown in Figure 2. Treatment with Daivobet^® ^also induced a highly significant reduction in CD3, CD4, CD8 and CD45RO positive T cells (Table 1).

Treatment with calcipotriol for three weeks reduced the epidermal thickness significantly (22%) compared to the vehicle treated group. The epidermal morphological parameters (extent of stratum corneum, stratum granulosum and parakeratosis) were also significantly influenced by calcipotriol treatment (Table 1). Although both the number of proliferating Ki-67 positive cells and the fraction of CK16 positive epithelium were reduced compared to vehicle (36% and 47%, respectively) these numbers did not reach statistical significance (Table 1). In addition, none of the T-lymphocyte subsets (CD3, CD4, CD8 and CD45RO) was significantly influenced by calcipotriol treatment (Table 1 and Figure 3).

Interestingly, neither calcipotriol nor Daivobet^® ^treatment induced any change in the fraction of CK10 expression epithelium.

### Psoriasis xenograft SCID mouse model

**Figure 4:**
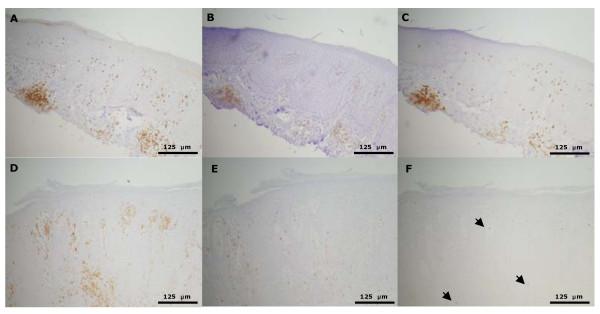
**Immunohistochemical stainings of a keratome biopsy before (A, B and C) transplantation and after (D, E and F) transplantation and treatment in the psoriasis xenograft SCID mouse**. Before transplantation the expression of CD3 (A), CD4 (B) and CD8 (C) is seen in follicular structures and diffusely distributed in the skin. However, the intensity of CD4 expression is slightly decreased compared to freshly excised and fixed psoriatic skin (data not shown). Five weeks after transplantation including a three week treatment with vehicle the expression of CD3 (D) expression in the skin seems to be increased. In contrast, CD4 (E) and CD8 (F; arrows) expression is down regulated after vehicle treatment. The skin from A, B, C, D, E and F is from the same donor.

At study end the mean epidermal thickness for the vehicle (n = 6) treated animals was 344 ± 123 μm, 236 ± 47 μm for calcipotriol (n = 7) treated animals (31% reduced epidermal thickness compared with the vehicle group) and 94 ± 49 μm for betamethasone diproprionate (BDP) (n = 4) treated animals (73% reduced epidermal thickness compared with vehicle group) (Table 2). This reduction only reached statistical significance for BDP when compared to the vehicle group. The effect on the biomarkers showed the same trends as observed in the patient study (Table 2). A comparison of the keratome biopsies before and after transplantation showed that the number of CD3 seemed to increase and the expression of CD4 and CD8 decreased (Figure 4). The dose of calcipotriol and BDP was well tolerated and no significant weight loss was observed. Two hours after the last application, the animals were bled and sacrificed. The serum levels of calcipotriol and BDP were analyzed and determined to be below the detection limit (data not shown).

**Table 2:** Immunohistochemical scores obtained from skin biopsies from xenografted SCID mice treated with vehicle, calcipotriol and betamethasone dipropionate (BDP).

	Vehicle	calcipotriol		Betamethasone		
	n = 5	n = 7	p-value vs. vehicle	n = 4	p-value vs. vehicle	p-value vs. calcipotriol
CD3 (number/mm^2^)	391.4 ± 152.1	393.7 ± 230.5	NS	134.7 ± 55.6	0.007	0.025
CD4 (number/mm^2^)	87.7 ± 110.7	67.6 ± 49.6	NS	5.8 ± 2.3	NS	0.016
CD8 (number/mm^2^)	32.9 ± 27.1	36.6 ± 42.3	NS	9.7 ± 6.6	NS	NS
CD45RO (number/mm^2^)	190.1 ± 191.1	172.1 ± 132.9	NS	103.3 ± 89.8	NS	NS
Ki-67 (number/mm)	439.0 ± 278.0	362.8 ± 255.2	NS	147.8 ± 189.4	NS	NS
CK10 (% area)	70.6 ± 21.0	73.0 ± 21.9	NS	75.7 ± 14.9	NS	NS
CK16 (% area)	19.5 ± 13.5	11.9 ± 13.4	NS	7.9 ± 9.7	NS	NS
Epidermal thickness (μm)	356.1 ± 121.4	225.9 ± 117.1	NS	98.1 ± 14.9	0.002	0.038

NS, not significant.

## Discussion

The objective of this study was to assess the clinical and biomarker responses of psoriasis patients treated with topical anti-psoriatic compounds in a plaque test study and to compare these data with those obtained in larger clinical studies. Furthermore, we wanted to use the biomarker data to compare biological effects in patients and the psoriasis xenograft mouse model.

In this psoriasis plaque test study of Daivobet^® ^ointment and the calcipotriol ointment we observed a significant clinical effect of both treatments compared to vehicle treated skin. After three weeks of treatment the TCS was reduced by 84% and 61% by Daivobet^® ^and calcipotriol ointment, respectively. These clinical effects of Daivobet^® ^and calcipotriol ointment are in good agreement with previously published data from larger clinical studies [4-6]. The time course of the clinical effects also matched those seen in other clinical studies, with a fast onset by Daivobet^® ^already showing significant clinical effect at day 4. As in other topical psoriasis studies a significant vehicle effect was observed, with a reduction of 37% in the total clinical score over three weeks by the ointment vehicle alone [7]. In this study a treatment arm with betamethasone diproprionate ointment alone was not included as the effects of betamethasone and Daivobet has been compared in another plaque test study. Using our standardised procedures these studies correlate well with regard to clinical effect. Epidermal thickness, proliferative activity (Ki-67 expression) and cytokeratin 16 expression are generally considered good markers of psoriasis activity [8,9]. In this study, these parameters also correlated well with the clinical score in the vehicle group (Figure 2).

Furthermore, a number of morphological parameters, such as the degree of parakeratosis, stratum corneum and stratum granulosum, are profoundly altered in psoriasis compared to normal skin. In this study the morphological changes correlated well with the clinical score and the psoriatic phenotype was strongly reduced by both the calcipotriol ointment and Daivobet^® ^(Table 1).

The effectiveness of calcipotriol in the treatment of psoriasis has primarily been attributed to its effects on epidermal proliferation and differentiation/keratinisation [10-12] although the effects on skin infiltrating T cells are relevant to its mode of action [13,14]. Calcipotriol has been shown to reduce CD3, CD4, CD8 and CD45RO positive T-cells in psoriatic skin [15].

There was a clear trend towards reduction of Ki-67, CK16 and T cell infiltration by the calcipotriol ointment in this study compared to the vehicle group, although, this reduction did not reach statistical significance (Table 1). The biopsies in this study were taken on day 22 and the biomarkers were therefore only scored at the end of the study. Considering the marked vehicle effect of the ointment vehicle observed on clinical score, it is likely that the biomarkers are also strongly affected by the vehicle treatment. This is supported by the finding that the patients with most pronounced vehicle effect on clinical score had normalised their biomarker profile (epidermal morphology, epidermal thickness, CK16 and Ki-67 expression) in the vehicle treated area (data not shown).

In other studies the calcipotriol ointment has shown significant reduction in biomarkers of epidermal proliferation, differentiation and lymphocyte infiltration [15-17]. However, these studies compared the biomarker profiles before and after treatment with the calcipotriol ointment and they did not assess the effects of the vehicle on the biomarker responses. Ointment vehicles, as used in these studies, are known to have a significant effect on the clinical score in psoriasis and most likely also on the biomarkers.

Cytokeratin 10 is a marker of normal epidermal differentiation and previous studies have shown an increase in the keratinocyte population expressing CK10 after treatment with calcipotriol when comparing to the pre-treatment levels [14,15,17]. In this study, we were not able to demonstrate an effect of Daivobet^® ^or calcipotriol ointment on cytokeratin 10 expression compared to vehicle. This is most likely due to the high vehicle effect and the lack of baseline data.

The psoriasis xenograft SCID mouse model is currently one of the most accepted and well characterized animal model for screening of novel anti-psoriatic compounds [3,18]. In the present study, lesional psoriatic skin was removed from volunteer donors suffering from chronic plaque psoriasis and grafted onto the back of immune deficient SCID mice. It has been debated which parameters could be used as endpoints in this model. Histological parameters such acanthosis and hyperkeratosis are generally accepted to be maintained during the study. However, it is controversial to what extent immunological parameters such as changes in T cell populations can be used [19].

To evaluate the effect of the calcipotriol ointment and BDP in this model, we tested the compounds topically and compared the results to the findings from the plaque test. The effect on the biomarkers showed the same trends as observed in the plaque test study. The epidermal thickness was significantly reduced following treatment with both the calcipotriol ointment and steroid and a clear trend in the reduction of the number of CD3, CD4, CD8 and CD45R0 positive cells was observed following treatment with steroid but not following treatment with the calcipotriol ointment. Statistically significant differences between the effects of the treatments were not seen on the biomarkers due to low number of animals in the *in vivo *study. We also observed a reduction of CK16 and Ki67 following both treatments. This indicates that the model is valid in regard to many parameters when evaluating the effect of antipsoriatic drugs topically. However, a general reduction of all cellular markers was observed in the grafts following the study in all groups. Furthermore, the total number of CD4 or CD8 positive cells was 10 - 30% of the number of CD3 positive cells. Since this was not observed to the same extent in the patient study, it indicates that CD4 and CD8 are heavily down regulated during the study. The loss of CD4 and CD8 receptors may be a consequence of extensive activation and exhaustion among the T cells in the graft (Figure 4). Even though CD4 positive cells can be depleted effectively in the model [20], this indicates that the immune cells in the grafts undergo severe phenotypic changes during the study. Thus, conclusions in regard to investigations of cellular biomarkers should be treated with caution as they could be misleading when investigated in the psoriasis xenograft SCID mouse model. In spite of these shortcomings, the xenograft mouse model is a useful preclinical psoriasis model that provides important information on the biological effect of anti-psoriatic treatments. On the other hand, the plaque test model clearly provides much more relevant data. This study has demonstrated that both clinical and biomarker results obtained from the psoriasis plaque test are in line with regular clinical studies. It is therefore an excellent method for obtaining early clinical "proof-of-concept" for comparing several treatments and for exploring the mechanism of action of topical anti-psoriatic treatments in the relevant patient setting.

## Conclusion

Our study demonstrates the feasibility of obtaining robust biomarker data in the psoriasis plaque test that correlate well with those obtained in other clinical studies. Furthermore, the biomarker data from the plaque test correlate with biopsy data from the grafted mice.

## Abbreviations

CK10: cytokeratin 10; CK16: cytokeratin 16; BDP: betamethasone dipropionate; SCID: severe combined immunodeficiency; TCS: total clinical score.

## Competing interests

PHK, LS, OH, VH, KK and MAR are employed by LEO Pharma A/S.

## Authors' contributions

PHK did the immunohistochemical evaluations, LS designed and conducted the animal studies, OH did the statistical analysis, VH organised the clinical study, KK and MAR participated in the design of the studies and interpreted the data. All authors read and approved the final manuscript.
